# Imaging of Metastatic Lymph Nodes by X-ray Phase-Contrast Micro-Tomography

**DOI:** 10.1371/journal.pone.0054047

**Published:** 2013-01-18

**Authors:** Torben Haugaard Jensen, Martin Bech, Tina Binderup, Arvid Böttiger, Christian David, Timm Weitkamp, Irene Zanette, Elena Reznikova, Jürgen Mohr, Fritz Rank, Robert Feidenhans’l, Andreas Kjær, Liselotte Højgaard, Franz Pfeiffer

**Affiliations:** 1 Niels Bohr Institute, University of Copenhagen, Copenhagen, Denmark; 2 Department of Physics, Technische Universität München, Munich, Germany; 3 Medical Radiation Physics, Lund University, Lund, Sweden; 4 Dept. of Clinical Physiology, Rigshospitalet, Copenhagen, Denmark; 5 Cluster for Molecular Imaging, University of Copenhagen, Copenhagen, Denmark; 6 Paul Scherrer Institut, Villigen, Switzerland; 7 Synchrotron Soleil, Gif-sur-Yvette, France; 8 European Synchrotron Radiation Facility, Grenoble, France; 9 Institute for Microstructure Technology, Karlsruhe Institute of Technology, Karlsruhe, Germany; University of Tennessee, United States of America

## Abstract

Invasive cancer causes a change in density in the affected tissue, which can be visualized by x-ray phase-contrast tomography. However, the diagnostic value of this method has so far not been investigated in detail. Therefore, the purpose of this study was, in a blinded manner, to investigate whether malignancy could be revealed by non-invasive x-ray phase-contrast tomography in lymph nodes from breast cancer patients. Seventeen formalin-fixed paraffin-embedded lymph nodes from 10 female patients (age range 37–83 years) diagnosed with invasive ductal carcinomas were analyzed by X-ray phase-contrast tomography. Ten lymph nodes had metastatic deposits and 7 were benign. The phase-contrast images were analyzed according to standards for conventional CT images looking for characteristics usually only visible by pathological examinations. Histopathology was used as reference. The result of this study was that the diagnostic sensitivity of the image analysis for detecting malignancy was 100% and the specificity was 87%. The positive predictive value was 91% for detecting malignancy and the negative predictive value was 100%. We conclude that x-ray phase-contrast imaging can accurately detect density variations to obtain information regarding lymph node involvement previously inaccessible with standard absorption x-ray imaging.

## Introduction

Breast cancer is the leading cause of death in cancer among women [1]. Early diagnosis and accurate staging of the disease are crucial for proper treatment and improved prognosis. It is of major importance whether the cancer is confined to the breast or has spread to the adjacent lymph nodes. The current state-of-the-art method to investigate the regional axillary lymph nodes is the sentinel node (SNL) technique [2]. Prior to the final surgical intervention, a quantity of radio-labeled colloid is injected into the breast, usually in the region around the tumor. The distribution of the radiotracer is visualized by a gamma camera image, revealing the lymph nodes that are draining the breast tissue with the tumor. These local lymph nodes, usually numbering between one and three, are called the sentinel nodes. The surgeon identifies the sentinel node with a small Geiger probe and removes the nodes for investigation by the pathologist. This is done either perioperatively or following the primary operation. If the nodes show histological metastatic deposits, an axillary dissection is performed [3].

The sensitivity and specificity of the SNL technique is generally high. However, the technique is not feasible for all breast cancer patients, and a non-invasive tool for identification of malignant lymph nodes would be of particular interest for non-SNL candidates. Suggested contra-indications for SNL are multifocal or multicentric lesions, previous axillary surgery, previous irradiation therapy, and neo-adjuvant chemotherapy [4]–[6], although large investigations regarding these contraindications are still needed.

Conventional imaging cannot accurately assess axillary lymph node involvement. Different imaging approaches have been evaluated for the use in axillary staging, and currently computer tomography and ultrasonography are the most widely used. However, no imaging modality has yet shown satisfactory sensitivity or specificity in this respect. Factors such as size and shape have been used to discriminate between malignant and benign lymph nodes, but these evaluation criterions have their obvious limitations.

A newer approach using dynamic contrast-enhanced magnetic resonance seems promising for evaluation of primary tumors for breast cancer patients after neo-adjuvant chemotherapy and has been evaluated as a surrogate marker for prediction of nodal status [7]. This indirect assessment of nodal involvement may however be associated with an unsatisfactory high false-negative rate.

The diagnostic value of F-18 -Fluorodeoxy-glucose-Positron Emission Tomography (F-18-FDG-PET) with or without diagnostic CT has also been evaluated as a tool for preoperative axillary staging [8], [9]. But again, a sensitivity around 60–70% seems unsatisfactory compared to SNL verified by histopathological examination.

Due to the high prognostic implication of axillary lymph node involvement, it is of paramount importance to identify a possible spread of disease for proper staging and thereby choice of treatment strategy. Especially false negative cases should be minimized for improved prognosis but also false positive cases, since removal of the whole axilla is associated with significant morbidity such as lymphedema in the affected arm and limitations of shoulder movement [10], [11]. Thus, the development of a safe pre-operative, non-invasive imaging technique to identify lymph node metastases would be of tremendous value. Such an imaging technique could spare the patient for axillary surgery if negative, and if positive, the primary tumor and the affected nodes could be removed during the same operation and proper treatment initiated faster.

Tiny density variations in human tissue are difficult or impossible to detect with conventional X-ray imaging, but they can be visualized by grating-based phase-contrast X-ray tomography [12]–[18]. Due to the wave-optical interaction of x-rays with matter, the contrast available with phase-contrast imaging is much higher than standard X-ray absorption imaging. Hence, the density variations caused by invasive cancer can be visualized. Here we report high-contrast biomedical phase-contrast imaging using synchrotron radiation [19]–[21] applied to human lymph nodes. The purpose of this study was, in a blinded manner, to investigate whether malignancy could be revealed by non-invasive x-ray phase-contrast tomography in lymph nodes from breast cancer patients.

## Materials and Methods

### Samples

Written informed consent was obtained from all participants and the study was approved by the regional scientific ethical committee. Seventeen formalin-fixed, paraffin-embedded axillary lymph nodes were used for the study, all from axillary dissection specimens from women with invasive ductal breast carcinoma. All lymph nodes were weighed, measured on the longest diameter and cut in half prior to formalin-fixation. One half of the formalin fixed-paraffin-embedded lymph node was used for the study and the other for routine histological examination.

Each sample was paraffin embedded in a small plastic tray following the standard protocol for histology slicing. Haematoxylin and eosin staining (H&E-staining) was performed according to routine procedures on 10 µm sections from all samples on the experimental half of the lymph node to verify the diagnosis. In addition, prior to sectioning and staining, each sample was imaged using phase-contrast tomography. For x-ray phase-contrast imaging the paraffin embedded lymph nodes were removed from their trays and brought to the synchrotron radiation facility.

### Phase-contrast Micro-tomography

**Figure 1:**
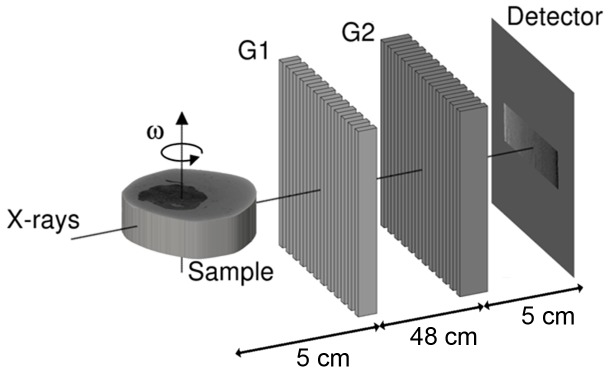
Experimental setup. Not to scale. The sample is placed in front of the interferometer as close as possible to the first grating. The distance between the two x-ray optical transmission gratings, G1 and G2 is dictated by the fractional Talbot order. The detector is placed immediately after G2. The phase-contrast images are collected for each rotation angle ω by scanning G1, along the transverse direction in four steps.

The x-ray imaging measurements were carried out at the ID19 beamline at the European Synchrotron Radiation Facility (ESRF), in Grenoble, France [22]. A grating interferometer was placed 150 meters from the wiggler source (see Figure 1). The principle and set-up of the x-ray grating interferometer has previously been described in detail [12], [23]–[27]. In this experiment we have used a monochromatic x-ray beam at 23 keV. The Si phase grating (G1, π-phase shifting) was placed after the sample, had period of 4.785 µm and a height of 29.5 µm and had been produced using photolithography and wet chemical etching [27]. The Au absorption grating (G2) was placed in front of the detector, had period of 2.400 µm and a height of 50 µm. It had been produced using soft X-ray lithography [28]. A FReLoN CCD detector with an effective pixel size of 14.9×14.9 µm^2^ with 1024×1024 pixels was used. The field of view was limited horizontally by the number of pixels of the detector, and vertically by the X-ray beam size; this resulted in a field of 15.3 mm width and 13.7 mm height.

For each tomographic dataset four phase step projections with an exposure time of 0.5 seconds each were collected for 701 angles evenly distributed over 360 degrees. This resulted in a total net exposure time of 24 minutes. Due to the large field-of-view four samples could be measured simultaneously. The data was reconstructed using a Hilbert-filter-based phase-contrast filtered back-projection algorithm [29]. A phase unwrapping procedure was applied prior to reconstruction to suppress cupping artifacts. For each sample the dataset included (depending on the size of the sample) 100–225 adjacent 14.9-µm-thick slices, thus providing a full 3D image of the sample.

### Data Analysis and Statistics

Each image was converted to DICOM format and imported into the Inveon Software (Siemens Medical Solutions, Inc., Knoxville, TN, USA) for analysis by two experts in Medical Imaging. The study was blinded and retrospective.

Since the technique was new and the images unlike prior absorption x-ray images, the experts who analyzed the scans first looked at a test set of three images and set their diagnosis, then they were given the correct diagnosis before completing the rest of the image analysis. This was done in order to know which features to look for in the benign and malignant lymph nodes respectively. The reference for metastatic disease was histopathology diagnosis set by a pathologist who was blinded to the x-ray phase-contrast results.

For comparison between groups for diameter and weight an independent-samples t-test was used. The statistical analyses were calculated using SPSS version 17.0 (SPSS Inc, Chicago, IL). P<0.05 was considered significant.

## Results

Twenty-three samples were visualized by phase-contrast micro-tomography. Due to technical challenges, as no experimental protocol was available before the experiment, some samples were excluded before the final image analysis. All samples were embedded in paraffin but they were removed from their trays in a way that was not optimized for the imaging procedure. For optimal phase-contrast imaging the sample embedding should be of convex shape and free of cracks. Establishment of this protocol lead to failed sample handling resulting in poor image quality of 6 samples. Therefore, seventeen lymph nodes from 10 patients were included in the final analysis. The age range of the 10 patients was 37–83 years (median age 60 years).

The 17 included lymph nodes were collected from 10 patients with clinical suspiciousness of nodal involvement and all underwent complete axillary resection. Of these 10 patients, 1 had no metastasis in the resected axillary specimen, 3 had 1–3 affected nodes, 3 had 4–6 affected nodes and 3 had more than 6 affected nodes.

Of the 17 included axillary lymph nodes, histological examination confirmed that n = 10 were infiltrated with invasive ductal breast carcinoma and n = 7 were without metastatic deposits. All lymph nodes with metastatic deposits that were included in the study had macro-metastases. The 10 lymph nodes with cancer infiltration had a weight of (4.1±3.3) g (mean ± standard deviation), and a diameter of (24±6.8) mm. The lymph nodes without malignancy had a weight of (0.6±0.4) g, and a diameter of (14±4.3) mm. The malignant lymph nodes had a significantly higher weight and larger diameter than the non-malignant lymph nodes (p = 0.015 and p = 0.005, respectively).

All of the 10 metastatic lymph nodes had a longest diameter that was above 10 mm, which is the criterion for considering the lymph node suspicious of malignancy on CT. Two lymph nodes were however close to 10 mm with the longest diameter of 12 and 13 mm respectively, which could be within the range of measurement inconsistence on CT images, and the lymph nodes could be considered non-malignant on conventional CT or not be detected at all.

**Figure 2:**
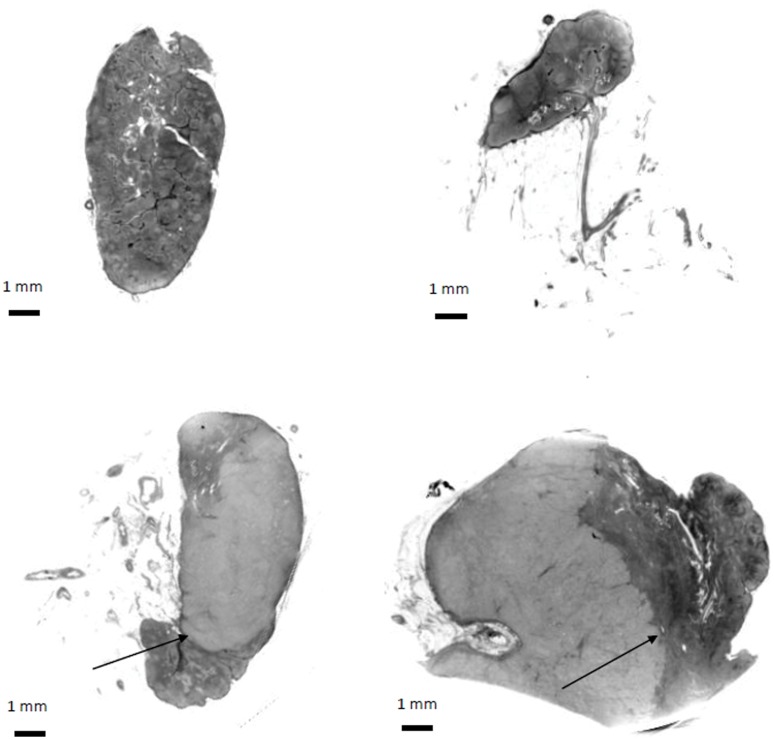
Phase-contrast tomography images of lymph nodes. Without (upper panels) and with (lower panels) metastatic deposits from patients diagnosed with invasive ductal breast carcinoma. The lymphoid follicles can easily be distinguished in the images of the benign lymph nodes. In the lower panels the invasive edge (arrows) clearly mark the border between the metastatic majority (lighter) part of the lymph node and the smaller part of the node with intact normal cells (darker).

The raw data and image reconstruction was carried out by experts in x-ray physics. The final images were analyzed by two experts in nuclear medicine and both the physicists and the physicians were blinded to the diagnosis of the lymph nodes. Each of the 2 experts in Nuclear Medicine who analyzed the images had 1 false positive result and no false negative results. The false positive sample was however different for the two experts. Thus, the inter-observer agreement was 88% (15 of 17 lymph nodes) for classification of the lymph nodes as malignant or benign. The diagnostic sensitivity of the image analysis was 100% and the specificity was 87%. The positive predictive value was 91% and the negative predictive value was 100%. The features that were characteristic of the benign lymph nodes, were an ordered structure with clearly visible lymphoid follicles (see figure 2 upper panels), whereas the malignant lymph nodes had features such as calcification, changed morphology, and a general disorganized appearance. From the example in figure 2 (lower panels) the border between the cancer-infiltrated and the non-infiltrated part of the lymph node can be seen with a clear gray level difference (cancer infiltration lighter) due to the changed density of the tissue caused by the cancer infiltration.

## Discussion

The axillary status is one of the most important prognostic factors for breast cancer patients. Therefore, correct assessment of nodal involvement is of outmost importance, and the sentinel node technique has evolved as a mandatory part in the evaluation and staging of breast cancer patients. However, the SNL is not always indicated, e.g. for patients undergoing neo-adjuvant chemotherapy, and other approaches for axillary assessment are warranted.

Using x-ray phase-contrast tomography we demonstrate here a high sensitivity (100%) as well as specificity (86%), positive predictive value (91%) and negative predictive value (100%) with no false-negative cases. In the non-metastatic lesions the lymphoid follicles were clearly visible whereas the metastatic lesions had lost this ordered morphology. This change in morphological characteristics was clearly visible upon image analysis. This is the first time that a non-invasive imaging modality has demonstrated the ability to visually identify morphological differences between malignant and benign lymph nodes. Accordingly, x-ray phase-contrast micro-tomography gives histological information in a non-invasive manner which allows for discrimination between benign and malignant lymph nodes.

In this study, micro-metastases were not analyzed, and the detection limit for identification of metastatic deposits in lymph nodes is so far not known for this imaging approach. However, given the high morphological information achievable with x-ray phase-contrast tomography, we believe that this technique is more powerful than imaging approaches otherwise available. In addition, it is questionable what the prognostic implication of micro-metastases is. It is known that intra-operative false-negative cases are often micro-metastases or isolated tumor cells. Two studies investigated the role of intra-operative false-negative cases. In these studies, 75% of the false-negative cases were micro-metastases or isolated tumor cells. There was no significant difference in recurrence rate between patients with true-negative SNL and patients with intra-operative false-negative SNL not undergoing axillary lymph node dissection [30], [31]. Thus, from the results of these studies it seems possible that the clinical significance of micro-metastases is limited.

The non-invasive diagnostic method proposed here, based upon x-ray phase-contrast imaging, can be used for identification of metastatic deposits in lymph nodes. Once developed into a clinical routine this x-ray phase-contrast imaging methodology could have a great impact on diagnosis and treatment in patients with breast cancer. The present sentinel node technique might then be replaced by a non-invasive preoperative x-ray phase-contrast evaluation of lymph node involvement in patients with breast cancer, and surgical intervention could be reduced to one operation, and perhaps more importantly, proper choice of treatment could be initiated faster.

The method of grating-based x-ray phase-contrast imaging has already been demonstrated using standard laboratory x-ray sources [16]–[17], [32]–[36], making its use in the clinic feasible. We believe that the images presented here will constitute the next step towards a new concept for imaging cancer and possibly other diseases. The implementation of phase-contrast x-ray imaging in a clinical environment could be envisioned as a high-resolution micro-CT for surgically removed tissue samples ex-vivo as an alternative to histology, or as an in-vivo full body CT.

In conclusion, we describe a new method using phase-contrast tomography that has a high sensitivity and specificity for non-invasive detection of lymph node metastases. The method has a high clinical potential and in the future may lead to non-invasive axillary staging of breast cancer patients.
